# Supplementary material for: Effect of Clinical and Laboratory Parameters on HDL Particle Composition
Source: Int J Mol Sci. 2023 Jan 19;24(3):1995. doi: 10.3390/ijms24031995 (PMC9916693; doi:10.3390/ijms24031995)
Supplement: Supplementary file 1 [file ijms-24-01995-s001.zip › ijms-2158094-supplementary.pdf]

Table S1: Correlation between serum lipid and apolipoprotein parameters and FA pattern of HDL

HDL FA pattern	Serum lipid and apolipoprotein profile							
	TC	TG	HDL-C	ApoAI	HDL-C/apoAI	LDL-C	nonHDL-C	Apo B
SFA	0.10*	0.01	-0.08	0.13	-0.13	0.12	0.13	-0.06
	-	-	-	-	-	-	-	-
UFA	-0.10	-0.01	0.08	-0.13	0.13	-0.12	-0.13	0.06
	-	-	-	-	-	-	-	-
MUFA	-0.19	-0.04	-0.10	-0.14	0.01	-0.12	-0.14	-0.04
	-	-	-	-	-	-	-	-
PUFA	0.04	0.04	0.17	-0.04	0.15	-0.04	-0.04	0.11
	-	-	-	-	-	-	-	-
LA	0.09	-0.09	0.03	0.08	0.05	0.11	0.08	0.19
	-	-	-	-	-	-	-	-
EPA + AA	-0.11	0.10	-0.21	-0.01	-0.20	-0.05	-0.01	0.07
	-	-	-	-	-	-	-	-
DHA	0.002	0.13	-0.02	0.01	0.27	0.05	0.01	0.20
	-	-	-	-	-	-	-	-
SFA/UFA	0.10	-0.01	-0.09	0.14	-0.13	0.14	0.14	-0.06
	-	-	-	-	-	-	-	-
SFA/PUFA	0.05	-0.04	-0.12	0.11	-0.14	0.11	0.11	-0.09
	-	-	-	-	-	-	-	-
**ACL**	-0.01	0.07	0.05	-0.04	0.11	-0.05	-0.04	0.16
	-	-	-	-	-	-	-	-

*: r Pearson Correlation, p value, -: not significant

Table S2: Serum lipid parameters and apolipoproteins of the two groups studied

	**Males**	**Females**	**p**
N	44	46	
Age, years	48.7 ± 12.8	50.3 ± 12.7	NS
TC (mg/dL)	174 ± 19	168 ± 21	NS
TG (mg/dL)	108 ± 34	97 ± 31	NS
HDL-C (mg/dL)	48 ± 11	50 ± 8	NS
LDL-C (mg/dL)	104 ± 22	101 ± 21	NS
non-HDL-C (mg/dL)	126 ± 20	119 ± 20	NS
apoAI (mg/dL)	139 ± 24	134 ± 21	NS
apo B (mg/dL)	80 ± 14	82 ± 19	NS
HDL-C/apoAI	0.3 ± 0.04	0.4 ± 0.05	NS
LDL-C/apoB	1.2 ± 0.2	1.2 ± 0.1	NS
non-HDL-C/apoB	1.5 ± 0.2	1.5 ± 0.2	NS

NS: not significant

Table S3: Effect of gender on the HDL phospholipid pattern in the healthy population

Phospholipids	Total			30-39 years		40-49 years		50-59 years		≥ 60 years	
	Males	Females	p	Males	Females	Males	Females	Males	Females	Males	Females
n	44	46		14	13	11	12	8	7	11	14
Total GPLs	42.01 ± 1.70	41.55 ± 2.12	NS	42.71 ± 0.81	43.19 ± 1.12	42.08 ± 1.75	41.64 ± 1.77	40.50 ± 2.12	40.60 ± 2.37	42.15 ± 1.69	40.45 ± 2.15*
PC	34.07 ± 1.76	32.27 ±2.36	<0.001	34.98 ± 1.85	33.11 ± 1.21**	33. 82 ± 1.43	33.89 ± 0.70	32.99 ± 2.00	30.61±2.58*	33.96 ± 1.35	30.96 ± 2.83**
LysoPC	3.02 ± 0.65	3.31 ± 0.77	NS	2.96 ± 0.65	2.88 ± 0.36	2.73 ± 0.37	3.08 ± 0.29*	3.26 ± 1.03	3.24 ± 0.95	3.20 ± 0.47	3.94 ± 0.89*
PE	1.02 ± 0.22	0.96 ± 0.27	NS	1.20 ± 0.15	1.22 ± 0.10	1.04 ± 0.16	1.09 ± 0.11	0.94 ± 0.28	0.71 ± 0.08*	0.83 ± 0.12	0.75 ± 0.26
PI	1.49 ± 0.50	1.26 ± 0.32	<0.05	1.44 ± 0.26	1.35 ± 0.22	1.72 ± 0.73	1.46 ± 0.27	1.41 ± 0.67	1.12 ± 0.33	1.38 ± 0.24	1.07 ± 0.31*
Rest GPLs	2.41 ± 1.78	3.75 ± 2.43	<0.01	2.13 ± 1.44	4.64 ± 1.83**	2.77 ± 1.87	2.12 ± 0.73	1.90 ± 0.64	4.92 ± 2.38*	2.78 ± 2.21	3.73 ± 2.62
Total ether GLs	4.90 ± 0.74	4.91 ± 0.76	NS	4.61 ± 0.72	4.87 ± 0.37	5.00 ± 0.78	5.48 ± 0.62	5.35 ± 0.82	4.69 ± 0.68	4.81 ± 0.53	4.59 ± 0.94
Plasmalogens	1.80 ± 0.24	1.74 ± 0.37	NS	1.82 ± 0.28	1.83 ± 0.23	1.80 ± 0.21	1.64 ± 0.14*	1.67 ± 0.16	1.55 ± 0.11*	1.88 ± 0.25	1.85 ± 0.60
Rest ether GLs	3.10 ± 0.82	3.17 ± 0.79	NS	2.79 ± 0.76	3.04 ± 0.37	3.20 ± 0.90	3.84 ± 0.73	3.68 ± 0.85	3.14 ± 0.62	2.93 ± 0.60	2.74 ± 0.88
Total SLs	7.32 ± 0.92	7.36 ± 0.89	NS	7.22 ± 0.69	7.37 ± 0.77	7.44 ± 1.09	7.42 ± 0.49	7.36 ± 1.14	7.22 ± 0.50	7.32 ± 0.95	7.32 ± 1.36
SM	6.35 ± 1.12	6.58 ± 1.08	NS	6.82 ± 0.77	7.07 ± 0.79	6.86 ± 0.98	7.03 ± 0.55	5.79 ± 1.27	6.13 ± 1.00	5.66 ± 1.09	5.96 ± 1.35
Rest SLs	0.97 ± 0.29	0.78 ± 0.71	NS	0.40 ± 0.36	0.30 ± 0.17	0.58 ± 0.39	0.39 ± 0.17	1.57 ± 0.51	1.09 ± 0.62	1.66 ± 1.34	1.36 ± 0.84
PC/SM	4.77 ± 0.54	4.97 ± 0.70	NS	5.11 ± 0.51	5.12 ± 0.52	4.89 ± 0.33	4.84 ± 0.41	4.40 ± 0.52	4.33 ± 0.23	4.49 ± 0.48	5.24 ± 0.98*

*p<0.05, **p<0.01: compared to males, NS: not significant

Table S4: Effect of gender on the HDL fatty acid pattern in the healthy population

Fatty acid pattern	Total			30-39 years		40-49 years		50-59 years		≥ 60 years	
	Men	Women	p	Men	Women	Men	Women	Men	Women	Men	Women
n	44	46		14	13	11	12	8	7	11	14
**SFA**	36.70 ± 6.91	36.84 ± 6.44	NS	32.51 ± 7.48	31.37 ± 6.41	37.01 ± 7.58	40.05 ± 4.09	38.06 ± 3.88	39.68 ± 6.30	40.71 ± 4.54	37.76 ± 5.31
**UFA**	63.30 ± 6.91	63.16 ± 6.44	NS	67.49 ± 7.48	68.63 ± 6.41	62.99 ± 7.58	59.95 ± 4.09	61.94 ± 3.88	60.32 ± 6.30	59.29 ± 4.54	62.24 ± 5.31
**MUFA**	8.40 ± 4.13	8.75 ± 5.60	NS	8.91 ± 4.04	13.41 ± 4.15**	7.73 ± 3.74	5.69 ± 4.84	8.84 ± 5.23	6.73 ± 4.78	8.08 ± 4.21	8.05 ± 5.39
**PUFA**	54.90 ± 6.66	54.41 ± 4.81	NS	58.58 ± 8.88	55.22 ± 7.57	55.26 ± 6.36	54.26 ± 3.82	53.10 ± 2.72	53.59 ± 2.08	51.21 ± 2.41	54.19 ± 3.46*
**LA**	19.12 ± 3.16	19.59 ± 2.45	NS	21.82 ± 3.17	21.91 ± 2.68	18.73 ± 2.03	18.00 ± 1.83	18.89 ± 2.21	18.98 ± 0.92	16.22 ± 1.63	19.08 ± 1.67***
**EPA+AA**	10.88 ± 1.84	10.61 ± 1.27	NS	11.31 ± 1.30	10.42 ± 1.33	10.92 ± 2.12	10.74 ± 1.18	9.80 ± 2.67	10.32 ± 0.98	11.10 ± 1.24	10.82 ± 1.47
**DHA**	3.55 ± 0.67	3.94 ± 0.76	<0.05	3.90 ± 0.53	4.41 ± 0.91	3.83 ± 0.48	4.01 ± 0.64	3.40 ± 0.62	3.74 ± 0.45	2.92 ± 0.57	3.54 ± 0.62*
**SFA/UFA**	0.60 ± 0.18	0.60 ± 0.16	NS	0.50 ± 0.18	0.47 ± 0.16	0.61 ± 0.21	0.68 ± 0.12	0.62 ± 0.10	0.67 ± 0.17	0.70 ± 0.13	0.62 ± 0.13
**SFA/PUFA**	0.69 ± 0.19	0.69 ± 0.17	NS	0.58 ± 0.21	0.60 ± 0.21	0.69 ± 0.22	0.74 ± 0.11	0.72 ± 0.07	0.74 ± 0.14	0.80 ± 0.11	0.70 ± 0.11*
**ACL**	16.35 ± 1.53	15.99 ± 1.58	NS	17.04 ± 1.36	16.79 ± 2.30	16.90 ± 1.68	16.13 ± 0.91	15.55 ± 1.02	14.76 ± 0.72	15.49 ± 1.33	15.76 ± 1.14

*p<0.05, **p<0.01,***p<0.001: compared to males, NS: not significant

Table S5: Serum lipid parameters and apolipoproteins of the premenopausal and postmenopausal females.

	**Premenopausal females**	**Postmenopausal females**	**p**
N	22	24	
Age, years	39.1 ± 5.5	60.5 ± 7.6	<0.001
TC (mg/dL)	166 ± 18	170 ± 24	NS
TG (mg/dL)	72 ± 20	101 ± 33	<0.01
HDL-C (mg/dL)	49 ± 7	50 ± 9	NS
LDL-C (mg/dL)	103 ± 20	100 ± 22	NS
non-HDL-C (mg/dL)	117 ± 20	120 ± 20	NS
apoAI (mg/dL)	123 ± 23	144 ± 25	<0.05
apo B (mg/dL)	86 ± 18	77 ± 19	NS
HDL-C/apoAI	0.4 ± 0.03	0.3 ± 0.06	<0.05
LDL-C/apoB	1.2 ± 0.1	1.3 ± 0.1	NS
non-HDL-C/apoB	1.4 ± 0.1	1.6 ± 0.2	<0.01

NS: not significant

Table S6: Effect of menopausal status on the fatty acid profile of esterified HDL lipids

Fatty acid pattern	Premenopausal	Postmenopausal	p
n	22	24	
Saturated fatty acids (SFA)	35.28 ± 7.28	38.28 ± 5.31	NS
Unsaturated fatty acids (UFA)	64.72 ± 7.28	61.72 ± 5.31	NS
Monounsaturated fatty acids (MUFA)	9.95 ± 6.20	7.64 ± 4.85	NS
Polyunsaturated fatty acids (PUFA)	54.77 ± 6.34	54.08 ± 2.86	NS
Linoleic acid (LA)	20.60 ± 2.76	18.66 ± 1.71	<0.01
Eicosapentaenoic + arachidonic acid (EPA + AA)	10.37 ± 1.12	10.81 ± 1.39	NS
Docosahexaenoic acid (DHA)	4.27 ± 0.84	3.36 ± 0.53	<0.01
Ratio			
SFA/UFA	0.56 ± 0.18	0.63 ± 0.13	NS
SFA/PUFA	0.76 ± 0.22	0.71 ± 0.12	NS
Average chain length (ACL)	16.51 ± 1.88	15.51 ± 1.07	NS

NS: not significant
